# Social and biological evaluation of antimicrobial resistance (SOBEAR) in rural India: a study protocol

**DOI:** 10.3389/fpubh.2024.1296382

**Published:** 2024-02-01

**Authors:** Matrujyoti Pattnaik, Ashish Kumar Nayak, Sonam Karna, Tanveer Rehman, Subrat Kumar Sahoo, Subrata Kumar Palo, Srikanta Kanungo, Jaya Singh Kshatri, Debaprasad Parai, Kamini Walia, Sanghamitra Pati, Debdutta Bhattacharya

**Affiliations:** ^1^Model Rural Health Research Unit, ICMR-Regional Medical Research Centre, Cuttack, India; ^2^Division of Epidemiology and Communicable Diseases, Indian Council of Medical Research, New Delhi, India

**Keywords:** social, biological, antibiotics, AMR, protocol

## Abstract

**Background:**

Antimicrobial resistance (AMR) has been one of the biggest global health threats in recent years, mostly in low- and middle-income countries, which requires urgent research using a multidisciplinary research approach. The use of large quantities of antimicrobial drugs inappropriately for humans, poultry and agriculture has been recognized as a leading cause of antibiotic resistance and the predominance of drug-resistance pathogens in the environment. This protocol aims to describe the use/misuse of antibiotics (ABs) in the community and evaluate clinical samples from healthcare settings to detect genes associated with antimicrobial resistance.

**Methods:**

We will conduct a community-level survey in different villages of the Tigiria block to assess knowledge and awareness on ABs and AMR. We will conduct in-depth interviews (IDIs) with doctors, pharmacists, nurses and drug sellers, as well as focus group discussions (FGDs) with ASHA and ANM workers who are involved in antibiotic supplies to the community. Quantitative data from the community survey and qualitative data of IDIs and FGDs will be linked and analyzed using statistical modeling and iterative thematic content analysis. Specimens (stool, urine, blood and wound/pus) will be collected from clinically diagnosed patients of different healthcare centers of Tigiria block. The samples will be cultured for bacterial isolation and antibiotic sensitivity testing. Genomic DNA will be isolated from positive bacterial cultures and sequenced using PCR to evaluate high-threat multi-drug resistance organisms (MDROs), screening of plasmid-mediated quinolone resistance (PMQR) genes, antimicrobial genes responsible for MDR and quinolone resistance-determining regions (QRDRs).

**Conclusion:**

This is the community-based protocol to evaluate the knowledge, attitudes, awareness and practices regarding ABs and AMR. The study protocol establishes a foundation for evaluating population-based prevalence and risk factors for AMR and MDROs in rural areas of the Odisha state, India.

## Introduction

Antimicrobial use in medicine, animal husbandry, veterinary practice and agriculture has reduced infection-causing agents by killing bacteria, viruses, fungi and parasites—hence the terms antibiotics, antivirals, antifungal, and anti-parasite were coined, respectively (1, 2). Antibiotics have been used successfully to arrest infection in various fields for an extended period. They have made it easy to manage and control infectious diseases, which contributed to the decline in cases of mortality and morbidity (3). In recent years, the advancement of antibiotics has put on risk global health through antimicrobial resistance (AMR) development in community and hospital settings (4). Globally, 700,000 deaths occur every year due to antimicrobial resistance (AMR) (4). If the current scenario continues, by 2050, AMR could result in over 10 million deaths annually and more than 100 trillion USD will be spent on healthcare (5). In response, the World Health Organization (WHO) has provided a standardized approach for the collection, interpretation and analysis of AMR in humans through a surveillance network called Global Antimicrobial Resistance and Use Surveillance System (GLASS). A worldwide global action plan (GAP) on AMR is also in place to handle the growing challenge and generate an international awareness program on AMR through effective education, training and communication (6).

AMR is a significant public health concern in India (7). Factors steering AMR growth in India are financial incentives for prescribing antibiotics, patient demand, and unregulated access to antibiotics (8). The prevalence of antibiotic usage from 2000 to 2018 in India increased from 46 to 67% (9). Additionally, antibiotic use in other sectors, such as food, agriculture and livestock, cumulatively increases the AMR burden in the country (10, 11). Indian Council of Medical Research (ICMR) established a National Programme on Antimicrobial Surveillance as part of the National Action Plan (12). This program primarily focuses on tertiary care centers to identify potential clinical pathogens.

Population or community-based survey accompanied by facility-based surveillance for diseases such as methicillin-resistant *Staphylococcus aureus* (MRSA), tuberculosis and HIV carriage has yielded more complete estimates to enhance associated risks for understanding, the prevalence and progress of improvisation of prevention programs (13–15). Different methods used in population or community-based surveys can be adapted to express AMR and multidrug resistance organisms (MDROs) colonization in the community. Implementing effective AMR surveillance systems to identify and measure AMR as it spreads is a crucial part of this method. Infections caused by MDROs such as MRSA, carbapenem-resistant *Enterobacteriaceae* (CRE) and extended-spectrum cephalosporin-resistant *Enterobacteriaceae* (ESCrE) can lead to disability or mortality, prolonged illness, and incur greater treatment costs than those caused by more susceptible organisms. In many countries, the recovery of bacteria from samples taken from patients with clinical illnesses is how AMR surveillance systems find these and other organisms. This technique is limited in many low- and middle-income countries due to lack of expertise, limited laboratory facilities, a shortage of supplies and reagents, and ineffective cross-sector data sharing. A less biased and more thorough perspective of the AMR phenotypes and genotypes circulating within a hospital or community can be obtained by quantifying human colonization with antimicrobial-resistant bacteria. When utilized in this way, colonization information can be a helpful addition to the regular AMR surveillance obtained from clinical samples.

This study will focus on a common protocol and methods for coordinating research to assess the community and hospital-based surveillance that will be followed for a period of 3 years and the different risk factors associated with AMR (misuse and overuse of antimicrobial drugs, poor access to quality and affordable medicines, lack of awareness, knowledge and enforcement of legislation) and associated with three MDROs of public health importance—MRSA, CRE, and ESCrE. The study will also target the screening of plasmid–mediated quinolone resistance (PMQR) genes, antimicrobial genes responsible for MDR and quinolone resistance-determining regions (QRDRs) in clinical pathogens through Polymerase Chain Reaction (PCR) technique. The study aims to (1) estimate the population-based prevalence of AMR and associated with MDRO, MRSA, CRE and ESCrE (2) the choice and distribution of antibiotics by patients and clinicians and their relative perception toward treatment (3) the public knowledge, awareness and common behaviors relevant to antibiotics use and antibiotic resistance and, (4) to assess the effectiveness of antimicrobial stewardship intervention among the stakeholders.

## Methods

### Study design

**Figure 1:**
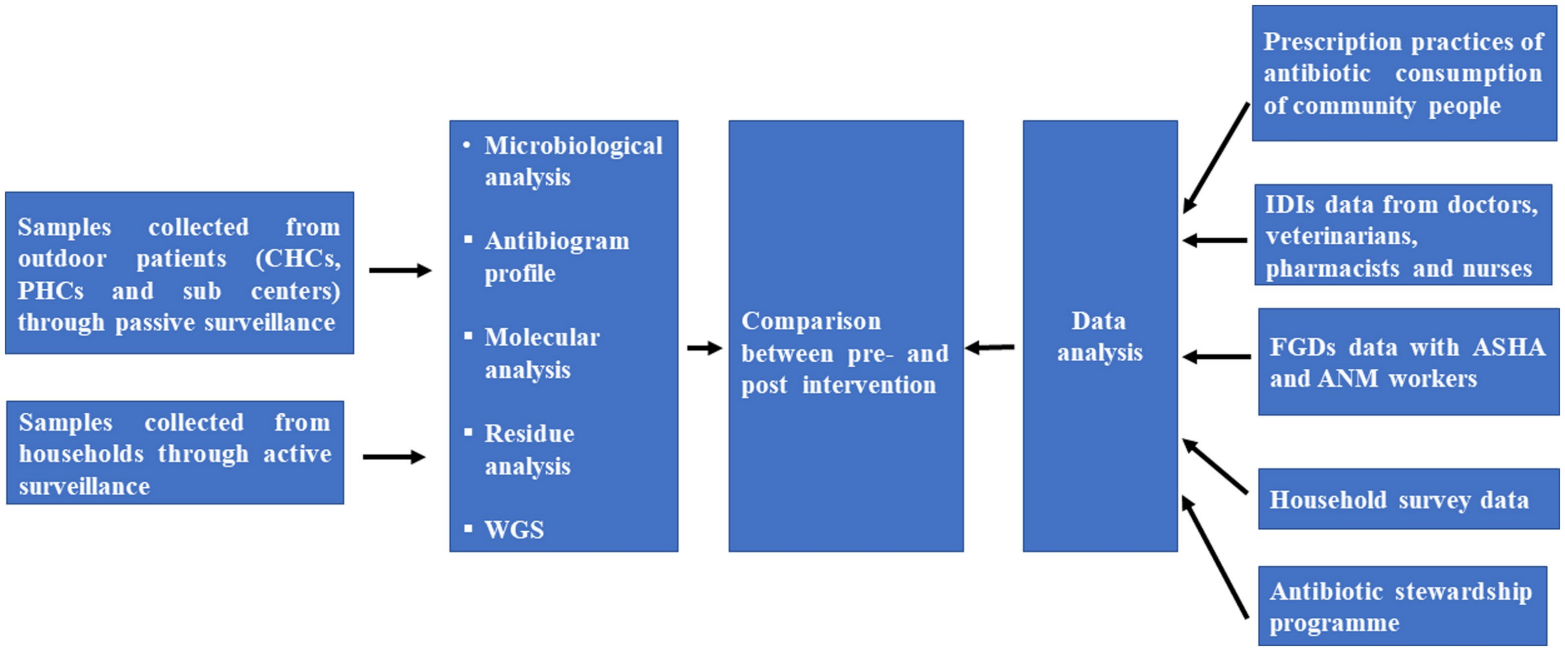
Main components of the community survey and surveillance system.

This is the study protocol for developing a surveillance system to detect AMR through active and passive surveillance of suspected cases. A mixed method involving both a qualitative and quantitative approaches will be conducted to determine the knowledge, attitude and practices of antibiotic usage, antibiotic prescribing and AMR. The primary components of the community survey and surveillance system are depicted in Figure 1.

### Study setting

**Figure 2:**
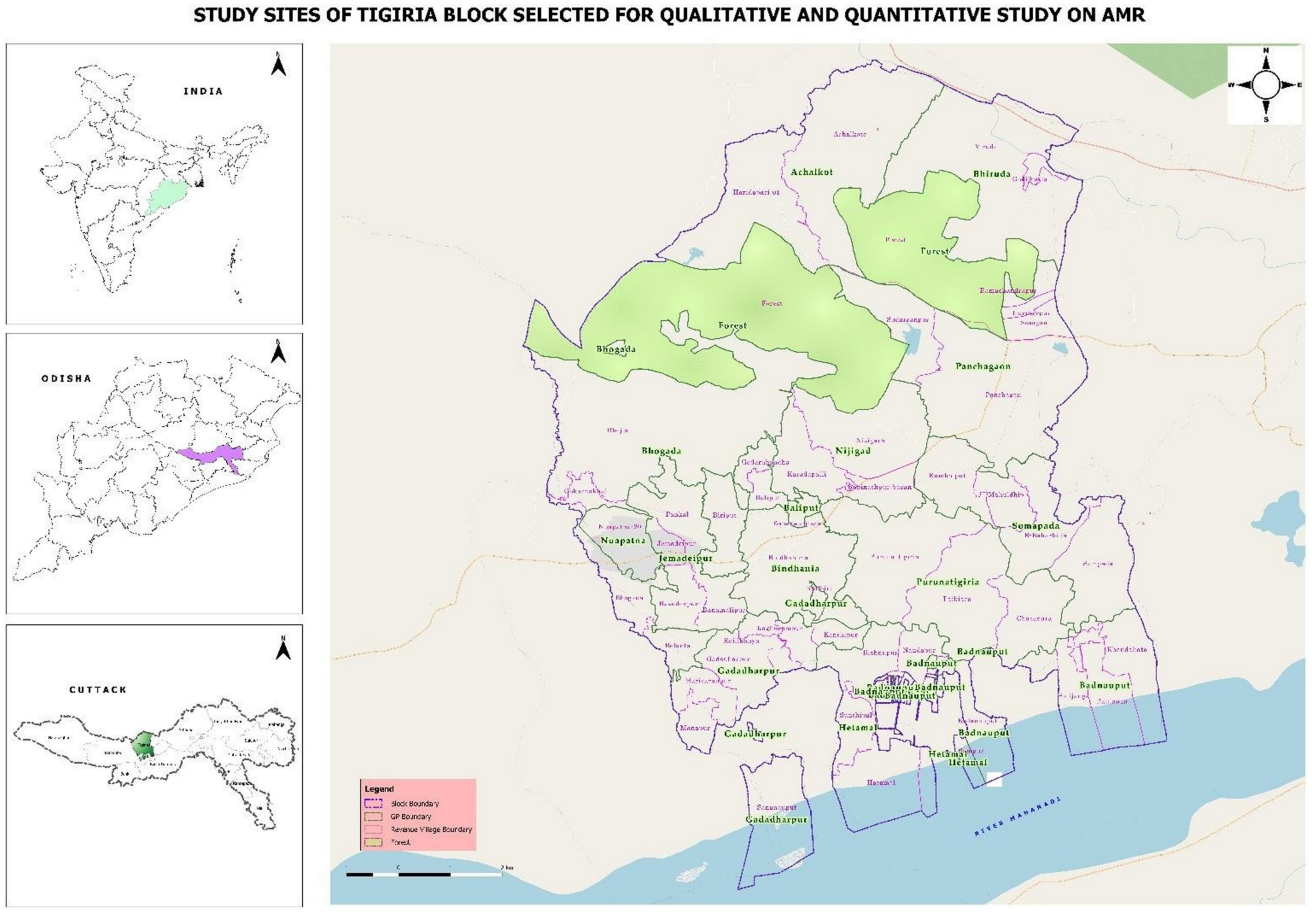
Geographical study location of Tigiria block with different villages (source: QGIS v3.18).

The study will be conducted in Tigiria block of Cuttack District, Odisha. Demographic Surveillance Site (DSS) of Tigiria block, Cuttack, caters to 50 villages and 14 panchayats, has been selected for the study (Figure 2). The DSS includes two Community Health Centres (CHC), four Primary Health Centres (PHC) and 14 sub-centres with a total population of 74,639.

### Study duration

We anticipate that the study will take 24–36 months to complete.

### Study population

The study has two target populations. They are individuals enrolled through active surveillance from households and those enrolled through passive surveillance from health facilities.

Eligibility criteria for enrollment of individuals from households through active surveillance are:

- 5 years of age and above
- With urinary tract infection (UTI), fever and diarrhea at the time of enrollment
- Must have been a resident of the village for at least 6 months

Eligibility criteria for enrollment of individuals from hospitals through passive surveillance are:

- 5 years of age and above
- Primary diagnosis of UTI, fever and diarrhea by the Medical Officer at the time of enrollment

For quantitative and qualitative survey on perceptions and determinants of antimicrobial use or misuse, the sampling of the population size and stakeholder mapping is given below.

### Sampling design and sample size

#### Household survey

A cluster sampling method will be employed, wherein 30 clusters or villages will be chosen by the probability proportional to size (PPS) method. From each cluster, 30 households will be selected for the community survey. The first house in the village will be determined by a random sampling method, and then a systematic random sampling method will be followed for the study in each cluster. Only one individual per household will be eligible for the community survey. Approximately 900–1,000 households will be selected for the quantitative study.

#### Sampling for hospital visit patients

The sample size was calculated for each syndrome (Diarrhea, Bacteremia, and UTI) separately using the formula 4pq/d^2^ where p is assumed prevalence, q is 1-p and d is relative precision. *E. coli* resistance to imipenem was considered for sample size calculation with 80% power, 5% alpha error, 20% relative precision and 10% non-response rate. The overall sample size for the AMR surveillance was calculated to be 7,316 (Diarrhea- 2830, Bacteremia-2830 and UTI- 1656).

#### Enrollment of quantitative community survey in villages

Once a person has agreed to participate post obtaining informed consent, field staff will administer a pre-tested questionnaire to collect data on socio-demographic, occupational exposures, healthcare services utilization, exposure history, antibiotic usage, antibiotic share, antibiotic resistance, AMR and hospitalization history.

#### Enrollment of hospital visit patients for laboratory surveillance

The field staff will approach patients from various health centers with due permission from doctors and pharmacists. Patients who meet the eligibility criteria must sign an informed consent form. Data and specimens will be collected from the patients only after obtaining the consent form. These data include socio-demographic characteristics, relevant exposure history and antibiotic history. The relevant applicable exposure record history and antibiotic history consists of the period of hospitalization previous to enrollment, records of procedures, systemic antibiotic exposure and outcomes of microbiological tests.

### Qualitative study for perception regarding antibiotic use

#### In-depth interviews with drug sellers and healthcare workers

We will select 15–20 drug sellers/pharmacies in different drug houses for IDIs to investigate their knowledge, attitudes, motivations and practices related to antibiotics and antibiotic prescription. As per the study requirement, we will select 10–15 doctors or clinician professionals, 5–8 pharmacists and 20–25 nurses from different hospitals (CHCs, PHCs, and health sub-centers)/private clinics in Tigiria block for qualitative IDIs to investigate their knowledge and attitudes on the sampling of specimens, antibiotics and AMR.

#### Community focus group discussions

Different community-based focus group discussions (FGD) will be conducted with ASHA and ANM working in Tigiria block, Cuttack, through maximum variation sampling. The broad themes of the FGD will be based on pathways of care, illness, experience of healthcare services and providers, prescription of antibiotics and AMR (Table 1). Purposive sampling will be used and participants will be enrolled till data saturation is achieved for the qualitative study design.

**Table 1:** Target sample size in rural areas of Tigiria block, Cuttack for data collection tool.

Sl. No.	Types of study	Per site study	Total nos. PHCs/CHCs/villages in Tigiria block	Study total
1	Community survey	30	30	900–1,000
3	IDIs with OPD patients	4–8	8–10	32–80
4	IDIs with doctors or clinicians	2–3	5	10–15
5	IDIs with drug sellers	2–4	7	14–28
6	IDIs with pharmacists	1	5	5
7	IDIs with health workers/nurses	1–2	5	5–10

IDI, In-depth interviews; OPD, Out-patient department.

#### Geospatial mapping

Using a GPS-enabled tablet, we will conduct geospatial mapping of antibiotics suppliers (hospitals, clinics, drug houses and informal drug sellers) in the local community of Tigiria block, Cuttack.

#### Collection of specimens for laboratory surveillance

Appropriate samples (stool/urine/blood) will be collected based on the primary diagnosis by the clinician.

#### Collection of stool and rectal swab specimens

Both enrolled households and hospital visit patients will be asked to provide stool or rectal swab specimen. Before taking samples, the collection staff or lab technician will provide specimen collection bottle to the participants and give instruct how to reduce the risk of contamination from the toilet surface, urine, and water. Within 3–4 h of collection, collection staff or lab technician will send specimen samples to the laboratory. Rectal swabs will be acquired by using a commercial kit and elution swabs by inserting a sterile swab into the rectum, spinning it, withdrawing it and depositing it into liquid amies transport media, which will be stored at 4°C until plating.

#### Collection of urine samples

Similarly, both household sites and hospital visit patients will be asked to provide urine sample. Before collection of samples, collection staff or lab technician will provide sample collection bottle and instructions to the participants to minimize the risk of contamination by toilet surface and toilet water. Around 10 mL of urine sample will be collected from patients. Due to the continual growth of bacteria *in vitro*, which alters the actual concentration of organisms, the samples will be delivered to the laboratory within 1 h of collection for bacteriological testing.

#### Collection of blood samples

After checkup and concerns by doctors, the hospital visit patients will be asked to provide blood sample. Bacteriological testing requires the use of whole blood. Around 2–3 mL of blood will be collected from patients. Serological procedures depend on serum extracted from blood. During the collection of samples, lab technician will take precautions for the participants to minimize the skin flora microorganism contamination. Blood samples will be collected and placed in Liquid Amies transport media, which will be stored at 4°C until plating. Within 2–4 h of collection, samples will be transported to the laboratory.

### Laboratory surveillance procedures

#### Microbiological and Molecular methods

##### Isolation and confirmation of clinical isolates

Standard operating procedures (SOPs) will be developed for the isolating, collecting, transporting and processing various specimens. When specimens arrive at the laboratory at Tigiria all specimens will be checked for proper labeling, safety, storage, quantity and quality of biological material. Delayed transport time, missing or incorrect labels and leakage upon arrival at the lab are some of the conditions under which specimens will be rejected from the list and documented accordingly. Specimens that are accepted will be put immediately onto distinct culture media that are specifically designed for each isolation, identification, and multidrug resistance organisms (MDROs). In most cases, all specimens (stool, urine and blood) will be inoculated on MacConkey and Nutrient agar. In some cases rectal swab samples will be inoculated on TCBS (Thiosulfate-Citrate-Bile Salts-Sucrose) agar media. For ESCrE, CRE, and MRSA culture of interest, Cysteine lactose electrolyte-deficient (CLED) agar or chromogenic agar media will be used for urine samples. Blood agar and chocolate agar will be included in the culture of the blood sample. After inoculating the specimen culture on different selective and differential media, plates will be incubated under standard conditions at 37°C overnight or 18–24 h. If growth is present, these isolates will be identified as bacteria and subjected to antibiotic susceptibility testing. To rapidly detect and determine the sequences of blaKPC, blaOXA-48, blaNDM, and CTX genes associated to carbapenem resistance in gram-negative bacteria, we will use Conventional Polymerase Chain Reaction to analyze all verified CRE strains from the culture (16).

##### Antibiotic susceptibility testing

All pure cultures on nutrient agar will be transferred to Muller Hilton agar plates with suitable antibiotics for Antibiotic susceptibility testing (AST). Kirby-Bauer disc diffusion method will be done for AST. AST will follow criteria provided by Clinical Laboratory Standards Institutes (CLSI) guidelines (17). Antibiotics that will be tested: Azithromycin, ciprofloxacin, levofloxacin, gentamycin, cefepime, ciprofloxacin, imipenem, tigecycline, meropenem, amikacin, ampicillin, ceftriaxone, tetracycline, ertapenem, amoxyclav, cotrimoxazole, nitrofurantoin, and nalidixic acid. The choice of antibiotics will be based on the culture samples and treatment of infections caused by all clinical isolates (both gram-positive and gram-negative) and as per the CLSI guidelines and Global Antimicrobial Resistance Surveillance System (18). AST results of all clinical isolates will be recorded in the form of resistance, sensitive or Intermediate Resistance as per CLSI guidelines (17) and further classified into mono-resistance, co-resistance and multi-drug resistance (MDR) (19). According to CLSI guidelines, ESBL will be detected phenotypically using the combined disc diffusion method with Muller Hinton agar and cefotaxime (30 g) and cefotaxime/clavulanic acid (30/10 g) and ceftazidime (30 g) and ceftazidime/clavulanic acid (30/10 g) and ceftazidime/clavulanic acid (30/10 g) and ceftazidime (17).

##### Molecular methods

**Figure 3:**
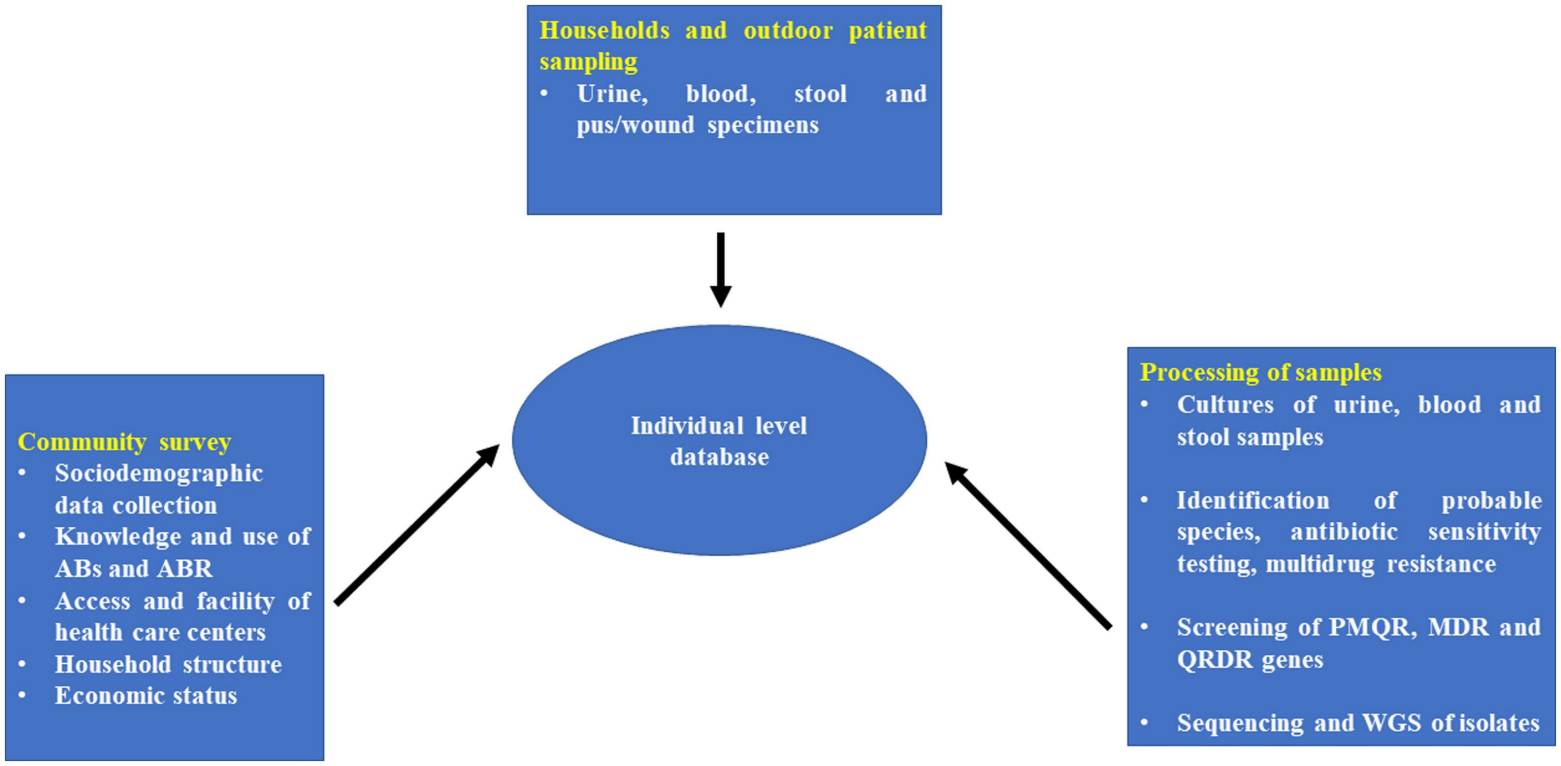
Individual patient linked database collection tool for rural areas/villages of Tigiria block.

After isolation and identification of pure culture, genomic DNA will be isolated from different bacterial species. Further genomic DNA will be used for screening of plasmid–mediated quinolone resistance (PMQR) genes, antimicrobial resistance genes responsible for MDR and quinolone resistance-determining regions (QRDRs) in enteric pathogens through PCR. During the study, *Enterobacteriaceae* (e.g., *E. coli*, *Klebsiella pneumoniae*, *Salmonella* spp., *Shigella* spp., and *Vibrio cholera*) and *S. aureus* isolates from blood samples collected at participating hospitals will be sent for sequencing and multilocus sequence typing (MLST) to create a library of sequence types for locally discovered infectious isolates in the study region (Figure 3).

### Laboratory monitoring

Quality control for the research laboratory conducting various microbiological tests (microbiological procedures like culture of clinical samples, isolation and identification of etiological agents and antimicrobial resistance) will be performed. To ensure the quality of the study, a recommended control strain will be plated alongside the specimen in each experiment. At regular intervals during the study period, microbiology monitoring staff will inspect all records kept in the laboratory for study and research purposes (e.g., specimen results, quality control logs, instruments logbooks), will periodically check on laboratory technical process and rightly follow-up SOPs, will observe the methodology process of research, will review the results of testing the simulated specimens and any needful actions. The research laboratory will always follow its usual SOPs for equipment maintenance and calibration during the study period. They will also regularly follow up on their standard biosafety and bio-securing procedure applicable during isolation, identification and sensitivity testing of antimicrobial-resistant microorganisms. Laboratory will follow the Good Laboratory Practice (GLP Guidelines) issued by WHO to maintain quality standards of the laboratory and keep it contamination free.

### Data management and analysis

Open Data Kit (ODK ver. 2023.3.6) will be used to make online data entries and to link data from all sub- studies. For sample tracing from field, as well as to maintain flow of samples within the laboratory, unique identities and bar codes will be employed. In every possible way, different electronic instruments like tablets, voice recorder will be used for data entry in both qualitative and quantitative study. Survey staff will entry the lab results into electronic data base. All survey data will be stored on password- protected tablets or computers and only the study team will have access to such data. The recorded information from qualitative research, such as focus groups and in-depth interviews, will be kept in a secure location. FGDs and IDIs will be translated and transcribed to facilitate analysis using MAXQDA (ver. 2022.4.2) software. Various themes and sub-themes will be generated using the inductive approach that will be analyzed by thematic framework. Integrating quantitative and qualitative data by specific mechanisms such as data transformation, joint display, convergent design, sequential exploratory design and data triangulation would enhance the robustness of the study. The information gathered from the numerous sub studies will be entered into data analysis tools such as SPSS (ver. 21.0) or STRATA (ver. 16.0). A comprehensive strategy will be developed for disseminating study findings to relevant stakeholders on a local and global scale, taking into account diverse communication channels such as peer-reviewed publications, conference presentations and involvement with mainstream and social media. In addition, focused outreach efforts will be made to policymakers, healthcare professionals, and community groups to ensure that the findings are accurate.

### Public and patient involvement

During the pilot project, community awareness programs and meeting with different stakeholders and community gatekeepers like gram panchayats (GP), self-help groups (SHG), local leaders will be held to bring together community people and health workers to discuss the findings and encourage community participation, which will help to increase antibacterial resistance stewardship. The public and patients will not be involved in the study’s conception or implementation.

### Ethical considerations

The study protocol has been approved by the Institutional Ethics Committee of Regional Medical Research Centre, Bhubaneswar (Ref No: *ICMR-RMRCB/IHEC-2019/034*) and will follow the National Ethical Guidelines for Biomedical and Health Research Involving Human Participants by the Indian Council of Medical Research (ICMR). The study will follow the ethical principles for medical research involving human subjects of the Declaration of Helsinki adopted by World Medical Association, 1964 which were last revised in October 2013. Samples will be collected from various CHCs and PHCs for research to be conducted at the Model Rural Health Research Unit (MRHRU), Tigiria block, in close coordination with State Health Department. Written informed consent will be taken from the participants before participating in the study and will be provided with PIS for their information. For children below 18 years of age, both ascent and consent forms will be obtained in the presence of their parents or guardians for enrollment in the study.

### Antimicrobial stewardship framework

**Figure 4:**
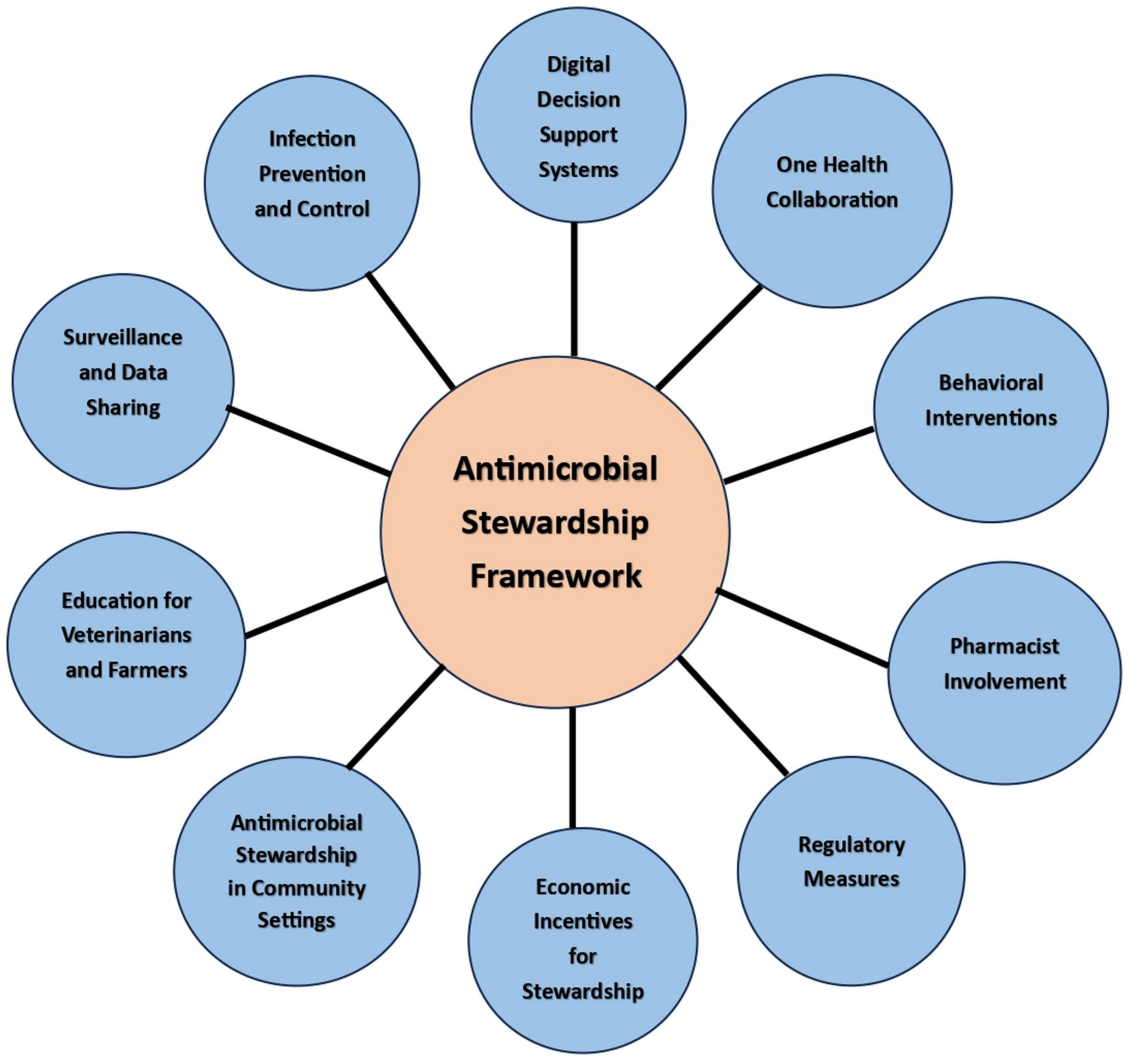
Antimicrobial stewardship framework proposed in our study.

Antimicrobial stewardship is a crucial approach to combating the rising problem of antimicrobial resistance and ensuring the appropriate and responsible use of antimicrobial agents. In the rural settings of India, where access to healthcare resources may be limited, it becomes even more important to implement a practical and effective antimicrobial stewardship framework (Figure 4).

1. Digital Decision Support Systems: Implement digital decision support tools integrated into electronic health records (EHR) to provide real-time guidance to healthcare providers on appropriate antibiotic selection, dosing, and duration. These systems can consider local resistance patterns, patient history, and individual risk factors.
2. One Health Collaboration: Foster collaboration between human healthcare, veterinary medicine, and environmental sectors to address antimicrobial resistance as a shared challenge. Implement joint surveillance and monitoring programs to understand the interconnection between human, animal, and environmental factors.
3. Behavioral Interventions: Conduct behavioral interventions and communication campaigns to raise awareness about antimicrobial resistance among healthcare providers, patients, and the general public. Promote responsible antibiotic use and the importance of completing prescribed courses.
4. Pharmacist Involvement: Involve pharmacists as key members of the antimicrobial stewardship team. They can play a vital role in reviewing prescriptions, suggesting appropriate alternatives, and providing patient education on proper antibiotic use.
5. Infection Prevention and Control: Strengthen infection prevention and control measures in healthcare facilities to reduce the incidence of infections. This can decrease the need for antibiotics and limit the spread of resistant organisms.
6. Surveillance and Data Sharing: Establish a national antimicrobial resistance surveillance network that enables data sharing among healthcare facilities, laboratories, and public health authorities. This data can guide targeted interventions and track progress.
7. Education for Veterinarians and Farmers: Provide education and training to veterinarians and farmers on responsible antibiotic use in agriculture. Encourage the adoption of alternative strategies to prevent and treat animal infections.
8. Antimicrobial Stewardship in Community Settings: Extend antimicrobial stewardship efforts beyond healthcare facilities to community settings. Engage community health workers to educate and monitor antibiotic use in remote or underserved areas.
9. Economic Incentives for Stewardship: Explore financial incentives for healthcare facilities that demonstrate successful antimicrobial stewardship practices. This can encourage institutions to invest in stewardship programs and commit to reducing resistance rates.
10. Regulatory Measures: Strengthen regulations around antibiotic use in healthcare and agriculture, including restrictions on the use of critically important antibiotics and measures to prevent over-the-counter sales of antibiotics.

### Antimicrobial stewardship interventions

Antimicrobial Stewardship (AMS) involves the careful and judicious selection, dosing, and duration of antimicrobial treatment to achieve the best possible clinical outcome for patients while minimizing any adverse effects and reducing the risk of future resistance (9). AMS empowers healthcare practitioners to ensure that each patient receives the most suitable antimicrobial agent at the appropriate dosage and duration. Providing optimal care to infected patients entails using the correct antibiotic, administered in the right dosage, and chosen with consideration for the least potential harm. The overarching objective is to curtail the excessive, inappropriate, and harmful use of antimicrobials. AMS aims to prevent the overuse, misuse, and abuse of antimicrobial agents across various sectors, including healthcare, veterinary medicine, and the environment. By implementing AMS, the progression of antimicrobial resistance can be mitigated. AMS course will be developed for the different stakeholders available in the Tigiria block.

### Development of modules

Antimicrobial stewardship modules will be developed using “One Health” approach for all healthcare care providers and veterinary officials in the Tigiria block. The modules will be developed for Medical Officers, Pharmacists, Veterinary Officers and ASHA/ANM (local healthcare providers). The modules will include various chapters on antimicrobial stewardship programs and its utility, catering to the needs of each stakeholder accordingly. The modules will be developed and printed for distribution at the stewardship training program of these stakeholders.

### Stewardship training of stakeholders

Experts from this field and from Indian Council of Medical Research (ICMR) institutes will organize a stewardship training program at the block level where the training will be provided to the Medical Officers, Pharmacists, Veterinary Officers and ASHA/ANM regarding antimicrobial stewardship. To address this growing health and financial threat of antibiotic resistance, all healthcare and other professionals must accept roles as frontline stewards. To protect our existing and future population, AMS can be achieved by prescribing medications appropriately and teaching patients and colleagues on how to use this increasingly scarce medical resource. We aim for long-lasting behavior change in an antibiotic prescription with AMS interventions.

### Evaluation of antimicrobial stewardship intervention

A baseline study will be conducted in the healthcare centers of Tigiria on doctors’ prescription of antibiotics to patients prior to the antimicrobial stewardship program. After the antimicrobial stewardship program, an endline study will be conducted to understand the antibiotics prescription practices of doctors following the stewardship intervention. This study will help us to evaluate the effectiveness of antimicrobial stewardship program in the rural region of India.

### Awareness programs in villages

Awareness programs will be conducted in all the 50 villages of Tigiria block with involvement of local healthcare providers and community heads regarding antimicrobial use, misuse and overuse. Medical officers and pharmacists will be involved to explain the concepts of antimicrobial resistance, its causes and effects to the community. Pamphlets will be made in local languages and distributed in the villages for spreading awareness on antimicrobial mis(use) and resistance. This Information Education Communication (IEC) will help to generate awareness among individuals and communities to develop and promote positive behavior for antimicrobial use.

## Discussion

The protocol methods summarize an approach to estimate prevalence and associated risk factors with relation to antibiotic resistance and MDROs in rural areas. The study is a conventional approach of tracking community settings and hospital visit patient on overall knowledge and awareness on antibiotics, AMR and MDROs. This study will generate data irrespective of gender, age, and other factors where it is often seen rural regions having high rate of MDROs but generally few surveillance data are available to guide prevention and control efforts. This study will also help people understand the problem of AMR and the way in which their use of antibiotics affects AMR emergence and the role by which they can play to control and change this trend. The study will also identify the geographical distribution of AMR pattern in the study population using GIS (Geographic Information System) tool.

The important findings from the study can be the utilization of current platforms for community-based studies, collection and simplify diagnostic procedure to detect antibiotic resistance and MDROs colonization in rural areas with diverse epidemiological characteristics to understand and control the spread of AMR and MDROs. An archive of AMR and MDROs study will provide opportunities to confirm policy, improve clinical practice and build the surveillance capacity of the pathogen in rural areas in near future. In certain cases, it is possible for bacteria carrying virulence genes, resistance genes, MLST patterns, and MDROs that are not typically associated with pathogenic bacteria to cause infections. Conversely, not all MDROs with similar virulence genes and MLST patterns as pathogenic bacteria will necessarily lead to infections in humans and their surrounding environment. The study will also include some advanced molecular research such as whole-genome sequencing (Oxford Nanopore) which intends to provide distinct depth coverage of genetic map for different antibiotic resistant genes and MDROs genes from isolated clinical samples. As a result, potential lead candidate genes will be discovered in the subsequent samples from various time points. Furthermore, using target sequencing of MDR gene function, it may reveal mutational changes that could be linked to antibiotic resistance.

The study presents several strengths, including the utilization of existing platforms for population-based research, streamlined diagnostic methods for detecting MDRO colonization, the inclusion of individuals from local communities and healthcare facilities, and the implementation across multiple locations with diverse epidemiological features to enhance comprehension of MDRO transmission. Categorizing colonizing isolates will facilitate assessing the impact of MDRO colonization on human health. The collection of MDROs will offer enhanced opportunities for further characterization and examination. Moving forward, the sites that have established baseline data can serve as a foundation for future investigations focused on genetic markers of resistance, microbiome analyses, and other endeavors aimed at advancing the global understanding of MDRO epidemiology. Meanwhile, laboratory surveillance of AMR provides valuable insights however; it is important to recognize its limitations, including potential selection bias, incomplete coverage, variability in testing methods, focus on specific pathogens, and exclusion of non-clinical settings. It is also not possible to explore the supply chain issues, or farming and animal husbandry, livelihood issues through the qualitative studies such as IDIs/FGDs. These limitations highlight the need for a multifaceted approach that combines laboratory data with other surveillance methods and considers the broader ecological factors influencing AMR.

In conclusion, establishing the prevalence of MDRO colonization within the population and identifying associated risk factors is essential for comprehending the extent of MDRO transmission. This knowledge will identify regions requiring coordinated mitigation strategies and could potentially lead to the development of a measure to gauge the effectiveness of prevention initiatives, free from the bias often seen in clinical infection data, which predominantly captures individuals already ill and potentially hosting more resilient bacteria. The outcomes of our study will enhance existing surveillance techniques for monitoring antimicrobial resistance and introduce novel perspectives on combatting this worldwide challenge.

## Ethics statement

The study protocol has been approved by the Institutional Ethics Committee of Regional Medical Research Centre, Bhubaneswar (Ref No: ICMR-RMRCB/IHEC-2019/034) and will follow the National Ethical Guidelines for Biomedical and Health Research Involving Human Participants by the Indian Council of Medical Research (ICMR).

## Author contributions

MP: Data curation, Formal analysis, Methodology, Project administration, Validation, Writing – original draft, Writing – review & editing. AN: Data curation, Formal analysis, Methodology, Project administration, Validation, Writing – original draft, Writing – review & editing. SoK: Data curation, Methodology, Project administration, Writing – review & editing. TR: Supervision, Validation, Writing – review & editing. SS: Methodology, Project administration, Writing – review & editing. SKP: Resources, Supervision, Writing – review & editing. SrK: Supervision, Validation, Writing – review & editing. JK: Supervision, Validation, Writing – review & editing. DP: Methodology, Resources, Validation, Writing – review & editing. KW: Conceptualization, Investigation, Supervision, Visualization, Writing – review & editing. SP: Conceptualization, Investigation, Supervision, Visualization, Writing – review & editing. DB: Conceptualization, Investigation, Supervision, Visualization, Writing – review & editing.
